# Nutritional ultrasound cut off for predicting complications and mortality in lung cancer patients: a multicenter longitudinal study from the VALONC project

**DOI:** 10.3389/fnut.2026.1876352

**Published:** 2026-09-17

**Authors:** Jose M. Romero-Márquez, María González-Pacheco, Rocío Fernández-Jiménez, Alba Carmona-Llanos, María Teresa Zarco-Martín, Pablo Rodríguez de Vera-Gómez, Fernanda Mucarzel-Suarez-Arana, Silvia García-Rey, Ana Sanz-Sanz, María Isabel Rebollo Pérez, María Luisa Fernandez-Soto, Vicente Mustieles, Araceli Muñoz-Garach, José Manuel García-Almeida

**Affiliations:** 1 Department of Endocrinology and Nutrition, Virgen de las Nieves University Hospital, Granada, Spain; 2 Foundation for Biosanitary Research of Eastern Andalusia—Alejandro Otero (FIBAO), Granada, Spain; 3 Faculty of Health Sciences, Atlantic-Mediterranean Technological University (UTAMED), Málaga, Spain; 4 Department of Endocrinology and Nutrition, Puerta del Mar University Hospital, Cádiz, Spain; 5 Instituto de Investigación e Innovación Biomédica de Cádiz (INiBICA), Hospital Universitario Puerta del Mar, Universidad de Cádiz, Cádiz, Spain; 6 PhD Program in Clinical Medicine and Public Health, University of Granada, Granada, Spain; 7 Department of Endocrinology and Nutrition, Virgen de la Victoria University Hospital, Málaga, Spain; 8 IBIMA Plataforma BIONAND, Instituto de Investigación Biomédica de Málaga, Málaga, Spain; 9 Department of Endocrinology and Nutrition, Quironsalud Málaga Hospital, Málaga, Spain; 10 Department of Medicine and Dermatology, Faculty of Medicine, University of Málaga, Málaga, Spain; 11 Department of Endocrinology and Nutrition, Jerez Universitary Hospital, Jerez de la Frontera, Spain; 12 Department of Nutrition and Food Sciencie, University of Granada, Granada, Spain; 13 Endocrinology and Nutrition Unit, San Cecilio University Hospital, Granada, Spain; 14 Granada Biosanitary Research Institute (ibs.GRANADA), Granada, Spain; 15 Department of Endocrinology and Nutrition, Virgen de la Macarena Universitary Hospital, Sevilla, Spain; 16 Nutrition Support Unit, Endocrinology and Nutrition Department, Hospital Universitari Vall d’Hebron, Barcelona, Spain; 17 Department of Endocrinology and Nutrition, Virgen del Rocío University Hospital, Seville, Spain; 18 Instituto de Biomedicina de Sevilla (IBIS), Seville, Spain; 19 Department of Endocrinology and Nutrition, Reina Sofía University Hospital, Cordoba, Spain; 20 Department of Endocrinology and Nutrition, Juan Ramón Jiménez University Hospital, Huelva, Spain; 21 Department of Medicine, University of Granada, Granada, Spain; 22 Consortium for Biomedical Research in Epidemiology and Public Health (CIBERESP), Granada, Spain; 23 Servicio de Radiodiagnóstico, Hospital Universitario Clínico San Cecilio, Granada, Spain; 24 CIBER Fisiopatología de la obesidad y Nutrición (CIBERObn), Instituto Salud Carlos III, Madrid, Spain

**Keywords:** dynapenia, GLIM criteria, malnutrition, morphofunctional assessment, nutritional ultrasound, rectus femoris, sarcopenia

## Abstract

**Introduction:**

Lung cancer remains a leading cause of cancer-related mortality, often exacerbated by malnutrition and muscle depletion that traditional weight-centric metrics may fail to identify. This study assessed the clinical and prognostic relevance of rectus femoris (RF) ultrasound as a practical bedside tool for evaluating muscle-related parameters and clinical outcomes.

**Methods:**

This multicenter longitudinal study involved 374 patients (114 females, 260 males) across 10 Spanish hospitals. Researchers performed ultrasonographic assessments of the RF, measuring cross-sectional area (RF-CSA), thickness (*Y*-axis), and circumference, alongside GLIM malnutrition criteria and PG-SGA. Statistical analyses included Random Forest algorithms, ROC curves, and Cox regression models adjusted for age, BMI, ECOG performance status, cancer type, and cancer stage to determine sex-specific associations with clinical outcomes.

**Results:**

Significant sexual dimorphism was observed; men exhibited larger muscle parameters, while women had higher subcutaneous fat. RF-CSA and RF-*Y*-axis showed the highest discriminative performance for GLIM-defined malnutrition, with AUCs of 0.706 and 0.713 in men and 0.814 and 0.804 in women, respectively. The corresponding RF-CSA cut-offs were 3.88 cm^2^ in men and 2.78 cm^2^ in women, whereas RF-*Y*-axis cut-offs were 1.09 cm in men and 0.95 cm in women. In men, RF-*Y*-axis was significantly associated with all-cause mortality (HR = 0.32, 95% CI: 0.15–0.71), whereas RF-CSA, RF-CIR, and RF-*X*-axis were associated with clinical complications. No significant associations with mortality or complications were observed in women. A moderate agreement was observed between GLIM and PG-SGA (weighted *κ* = 0.51), suggesting that these criteria capture overlapping but not identical dimensions of malnutrition.

**Discussion:**

Rectus femoris ultrasound may provide objective, sex-specific clinical information that complements the GLIM framework and traditional anthropometric assessments. By identifying clinically relevant muscle waste, this bedside tool allows for the early detection of high-risk patients, potentially directing timely nutritional and rehabilitative interventions to improve survival and treatment tolerance in lung cancer.

## Graphical abstract

**Figure:**
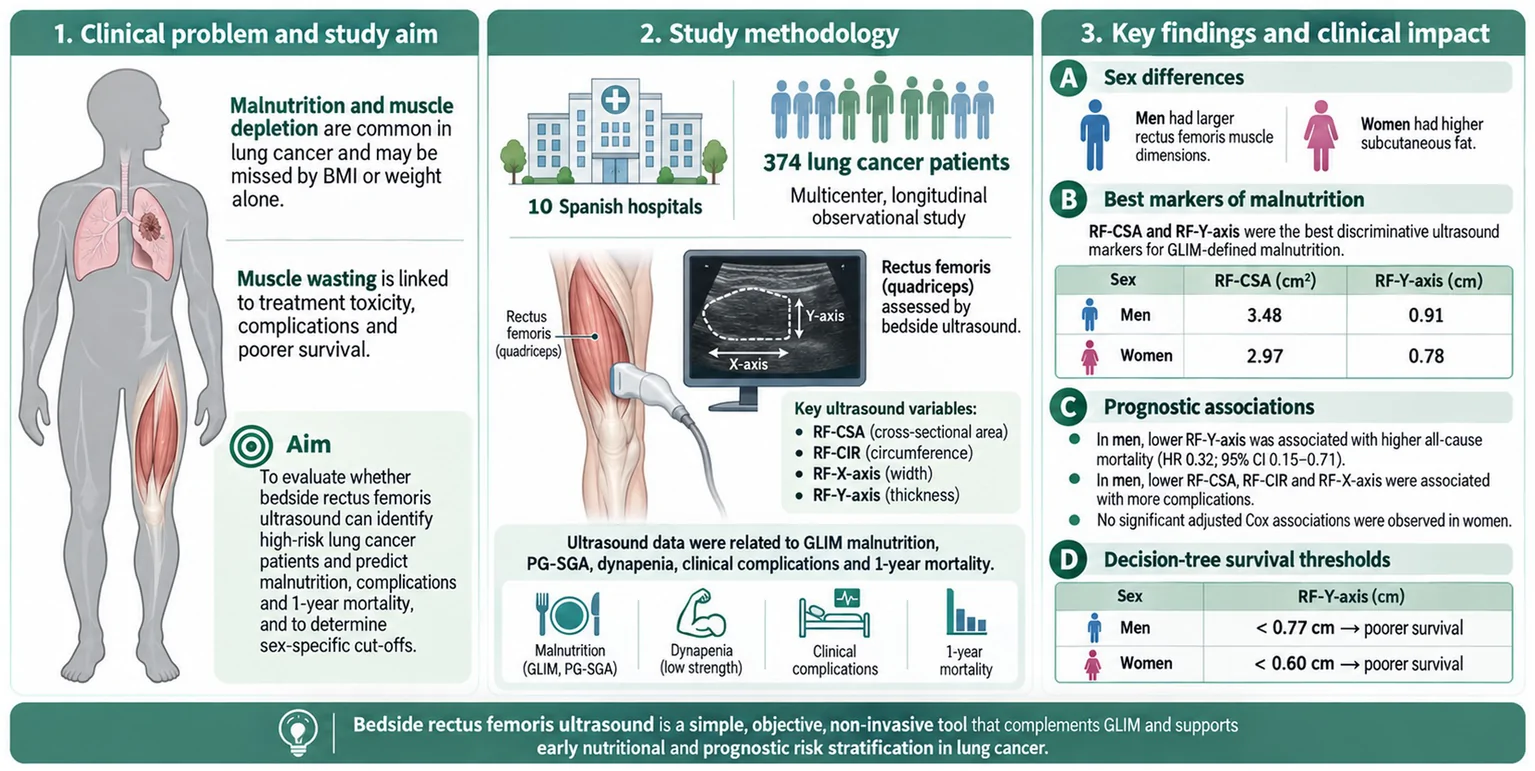
This figure was generated using ChatGPT – OpenAI, ChatGPT platform; GPT-5 model and NotebookLM Plus – Google LLC, NotebookLM Plus platform; powered by Google’s Gemini family of models. The input prompts can be found in Supplementary File 2.

## 1 Introduction

Lung cancer remains one of the most prevalent malignancies globally and continues to be a leading cause of cancer-related death, reflecting both its aggressive biological behavior and the considerable burden of treatment-associated complications (1–3). Despite substantial advances in management, including the integration of chemotherapy, radiotherapy, and immunotherapy, clinical outcomes are still unfavorable for many patients (4, 5). The need for prognostic indicators that go beyond tumor-related features alone is highlighted by this persistent deficiency. As a result, host-related determinants of outcome have received more attention, especially those that affect treatment tolerance, recovery potential, and survival in patient populations frequently impacted by functional decline and nutritional deterioration (6).

Alterations in body composition, especially reductions in skeletal muscle mass and function, have been consistently associated with adverse clinical outcomes across diverse diseases and are particularly prominent in oncology (7). In lung cancer, muscle wasting is associated with increased treatment toxicity, higher rates of postoperative recurrence, prolonged recovery periods, and reduced survival, even when body weight appears stable due to concomitant adiposity gain (8, 9). These findings highlight the limitations of traditional anthropometric metrics and weight-based screening methods, which may fail to detect clinically relevant muscle depletion. In this context, international nutrition guidelines increasingly recommend the systematic assessment of lean tissue within a comprehensive nutritional evaluation, recognizing skeletal muscle as a key determinant of metabolic flexibility, physical function, and prognosis (10).

Even though cross-sectional imaging modalities like computed tomography and magnetic resonance imaging can accurately quantify the quantity and quality of muscle, their routine use for body composition assessment is constrained by cost, accessibility issues, and, in the case of CT, cumulative radiation exposure (11). In this regard, muscle ultrasound has garnered attention as a practical bedside substitute for evaluating skeletal muscle in clinical practice that is non-invasive, affordable, and repeatable. Measures derived from ultrasound, such as muscle thickness, cross-sectional area, and echogenicity, support the assessment of tissue quality and muscle mass. There is mounting evidence that these measures have acceptable validity and reliability when compared to other established imaging techniques (12). Because of its accessibility, functional significance, and sensitivity to disease-related muscle atrophy, the quadriceps muscle group, in particular, the rectus femoris, has emerged as a preferred location within this method (12, 13). In fact, quadriceps ultrasonography may offer clinically significant prognostic information, particularly in lung cancer. Reduced physiological reserve, frailty, and malnutrition are known to affect treatment tolerance and survival given that smaller rectus femoris dimensions are likely to reflect muscle depletion linked to these conditions (14). Consistent with this rationale, muscle ultrasound–based evaluation has demonstrated prognostic relevance in other solid malignancies, such as head and neck cancer, where reduced rectus femoris cross-sectional area has been independently associated with higher odds of malnutrition and sarcopenia and with poorer survival outcome (15). Therefore, ultrasound allows for repeated longitudinal assessment of muscle status throughout the course of the disease and treatment, which may capture dynamic changes like acute muscle loss during oncological therapy, in contrast to retrospective evaluations based on static imaging (12, 13, 16). From a nutritional and clinical perspective, adding quadriceps ultrasound to routine evaluation could help identify patients at higher risk earlier, supplement current frameworks for nutritional risk and malnutrition, and direct focused supportive interventions. Taken together, these considerations suggest that rectus femoris ultrasound may have prognostic relevance in lung cancer and warrants further investigation within comprehensive, patient-centered assessment models.

Therefore, the aim of the present study was to evaluate the prognostic value of rectus femoris ultrasound in predicting malnutrition, treatment-related complications, and mortality in a large multicenter cohort of patients with lung cancer, and to determine sex-specific diagnostic and prognostic cut-off points.

## 2 Materials and methods

### 2.1 Study design

This multicenter, longitudinal, observational study of routine clinical practice was conducted by the VALONC group (valoración morfofuncional en pacientes oncológicos con cáncer de pulmón) within the Departments of Endocrinology and Nutrition of 10 hospitals in Spain between 2023 and 2024. Participating centers included Hospital Virgen de la Victoria (Málaga), Hospital de Jerez (Cádiz), Hospital Puerta del Mar (Cádiz), Hospital Universitario Virgen de las Nieves (Granada), Hospital Universitario San Cecilio (Granada), Hospital Universitario Virgen del Rocío (Seville), Hospital Universitario Virgen Macarena (Seville), Hospital Universitario Juan Ramón Jiménez (Huelva), Hospital Universitario Reina Sofía (Córdoba), Hospital Universitario Vall d’Hebron (Barcelona). The study received approval from the local ethics committee prior to initiation on 25 July 2023 (protocol number 1085-N-23).

### 2.2 Patient selection

A total of 374 patients diagnosed with lung cancer at different stages were enrolled in the study. Diagnoses were confirmed through review of medical records and pathological examination, with biopsy specimens classified by pathologists according to histological characteristics in line with the World Health Organization Classification of Tumors guidelines (2016). Patients were recruited within the first 2 weeks of initiating oncotherapy or other systemic treatments, and all participants were followed for a minimum of 1 year. Follow-up consisted of scheduled clinical assessments at diagnosis, 6 and 12 months during the first year. Patients were eligible for inclusion if they had a confirmed diagnosis of lung cancer and were evaluated within 2–4 weeks after diagnosis, prior to undergoing surgery (preoperative assessment) or initiating radiotherapy/chemotherapy treatment. Eligible participants were between 18 and 85 years of age and provided written informed consent. Patients were excluded if they were unable to adequately complete the clinical and analytical assessments required by the study protocol, if they were not expected to complete follow-up, or if their estimated life expectancy was less than 3 months.

### 2.3 Demographic and anthropometric variables

Demographic and anthropometric data were collected, including age (years), sex (male/female), body mass index (BMI, kg/m^2^), and body weight (kg).

### 2.4 Clinicopathological variables

Clinical variables collected included cancer type (non-small cell lung cancer [NSCLC], small cell lung cancer [SCLC], mesothelioma, or other thoracic malignancies), TNM stage (I–IV), receipt of chemotherapy (yes/no), and the occurrence of complications (yes/no), specifically dermatitis, dysphagia, mucositis, and asthenia. Data on unplanned mortality (yes/no), was also recorded. Functional status and quality of life were assessed using the Eastern Cooperative Oncology Group (ECOG) performance status scale, ranging from 0 (asymptomatic, fully active) to 5 (death).

### 2.5 Biochemical variables

Biochemical markers were assessed, including albumin (g/dL), prealbumin (mg/dL), total protein (g/dL), total cholesterol (mg/dL), creatinine (mg/dL), estimated glomerular filtration rate (mL/min), urea (mg/dL), glucose (mg/dL), C-reactive protein (CRP, mg/dL), thyroid-stimulating hormone (TSH, μIU/mL), 25-hydorxyvitamin D (ng/mL), and glycated hemoglobin (HbA1c, %).

### 2.6 Body composition measurements

Body weight was measured to the nearest 0.1 kg using a calibrated scale, with participants standing barefoot in light clothing. Height was assessed with a stadiometer (Seca, Hamburg, Germany). Ultrasonographic assessment of the quadriceps rectus femoris (RF) was performed in all participants using a 10–12 MHz linear multifrequency probe (Mindray Z60, Madrid, Spain), with subjects positioned supine (12). Measurements were obtained without probe compression at the junction of the lower third between the anterior superior iliac spine and the superior pole of the patella. Anteroposterior muscle thickness, muscle circumference, and cross-sectional area were recorded. The probe was positioned perpendicular to both the longitudinal and transverse axes of the RF to obtain rectus femoris cross-sectional area (RF-CSA), rectus femoris circumference (RF-CIR), transverse (*X*-axis) and anteroposterior (*Y*-axis) diameters, as well as leg subcutaneous adipose tissue (L-SAT). Every ultrasonographic assessment was carried out by a sanitary personal who had previously received training in the method. Three measurements were made for each parameter, and the analysis was based on the mean of these values.

In order to measure abdominal adipose, the probe was placed halfway between the xiphoid process and the umbilicus. Preperitoneal (visceral) adipose tissue (VAT), superficial subcutaneous abdominal fat (S-SAT), and total subcutaneous abdominal fat (T-SAT) were measured in centimeters at this site. T-SAT, L-SAT, and VAT were added up to determine global adipose tissue (GAT), which was then divided by the height to determine the global adipose tissue index (GATi).

### 2.7 Assessment of nutritional status and definition of clinical outcomes

The Global Leadership Initiative on Malnutrition (GLIM) criteria were used to diagnose malnutrition (17). At least one phenotypic and one etiologic criterion had to be present at the same time for malnutrition to be diagnosed. According to this framework, etiologic criteria included decreased food intake or assimilation and the existence of disease burden or inflammation, while phenotypic criteria included inadvertent weight loss, low BMI, and decreased muscle mass. Similarly, a dietary survey was used to measure decreased food intake, and each patients 24-h recall was collected via an online platform created by the University of Valladolid (18). Additionally, laboratory measurements like CRP, albumin, and prealbumin were used as supportive proxy indicators to assess inflammatory status. Lastly, to differentiate between moderate and severe malnutrition, diagnostic thresholds were used. A BMI of less than 20 kg/m^2^ for those under 70, a fat-free mass index (FFMI) of less than 22 kg/m^2^ for those over 70, an FFMI of less than 18.5 kg/m^2^ for those under 70 with weight loss of more than 10%, or a BMI of less than 20 kg/m^2^ for those over 70 with weight loss of more than 10% were all considered indicators of moderate malnutrition. The Patient-Generated Subjective Global Assessment (PG-SGA) was used concurrently to assess nutritional status and classify participants as well-nourished, moderately malnourished, or severely malnourished (19).

For the diagnosis of sarcopenia, reduced muscle mass and strength were evaluated using sex-specific cut-off values based on the revised criteria of the European Working Group on Sarcopenia in Older People (EWGSOP2). According to Cruz-Jentoft et al. (20), sarcopenia was defined as low handgrip strength together with low appendicular skeletal muscle mass index (ASMI), using thresholds of <27 kg for handgrip strength and <7.0 kg/m^2^ for ASMI in men, and <16 kg for handgrip strength and <5.5 kg/m^2^ for ASMI in women. In the same way, dynapenia was defined as a mean handgrip strength of less than 27 kg for men and less than 16 kg for women. Furthermore, according to the criteria outlined by Sánchez-Torralvo et al. (21), dynapenia was further stratified using age- and sex-specific reference standards for Spanish individuals. According to this method, dynapenia in men was defined as handgrip strength values below 34.7 kg for those under 45, below 35.5 kg for those between 45 and 59, and below 28 kg for those 60 years of age or older. Additionally, the corresponding thresholds for women were below 18 kg for those under 45, below 16.2 kg for those between 45 and 59, and below 12.6 kg for those 60 years of age or older.

The ASMI was used to assess muscle mass status. The ASMI cut-offs of <7.0 kg/m^2^ for men and <5.5 kg/m^2^ for women were used to define low muscle mass in accordance with EWGSOP2 consensus criteria. Lastly, dynapenia without low muscle mass was regarded as a separate clinical category. Individuals who met the handgrip strength criteria for dynapenia but did not meet the ASMI thresholds for low muscle mass were classified as having dynapenia without low muscle mass, whereas those with both low muscle mass and reduced muscle strength were classified separately.

### 2.8 Statistical analysis

Continuous variables are expressed as mean ± standard deviation (SD), and categorical variables as absolute and relative frequencies [n (%)]. Missing data were handled using the MissForest algorithm. The imputation process was performed on variables with less than 30% missingness, while variables exceeding this threshold were excluded from the analysis. The performance of the imputation was assessed using the out-of-bag (OOB) error. All analyses were stratified by sex. The normality of continuous variables was assessed using the Shapiro–Wilk test. Comparisons between groups were conducted using Welch’s *t*-test or the chi-squared test, as appropriate. *p*-values and odds ratios (ORs) with 95% confidence intervals (CIs) were presented. Age and BMI were considered in all models. Agreement between GLIM and PG-SGA classifications was assessed using weighted Cohen’s kappa coefficient, considering that both instruments may capture overlapping but not identical dimensions of malnutrition (weighted *κ* = 0.51). A Random Forest algorithm was used in machine learning analysis to identify the most relevant variables for classifying low muscle mass as defined by dynapenia and malnutrition based on GLIM criteria. The Gini index’s mean decrease was used to rank the variables’ importance; higher values indicated a greater contribution to the model’s performance. Cox regression models for mortality and complication outcomes were adjusted for age, BMI, ECOG performance status, cancer type, and cancer stage. In addition, decision tree-based models were developed to stratify mortality risk using ultrasound parameters in men and women. Kaplan–Meier survival curves were generated according to baseline nutritional ultrasound categories (low vs. high), and differences between groups were assessed using the log-rank test. All analyses and graphical representations were performed using R v.3.5.1 software (Integrated Development for R, RStudio, PBC, Boston, MA, United States). Statistical significance was set at a *p*-value of <0.05.

## 3 Results

### 3.1 Baseline characteristics and ultrasound characteristics measurement of the population included in the study

Table 1 summarizes the baseline characteristics of the study population according to sex. Male patients were significantly older than females (*p* < 0.001) and had a higher BMI (*p* = 0.018). Higher HbA1c levels were observed in males, although this difference did not reach conventional statistical significance (*p* = 0.051). Regarding lipid profile, males had significantly lower total cholesterol levels compared to females (*p* < 0.001). Males also showed higher urea (*p* = 0.001) and creatinine levels (*p* < 0.001), along with a lower estimated glomerular filtration rate (*p* = 0.012). Finally, radiotherapy was more frequently administered to males (*p* = 0.009), and mortality was also higher in males than in females (*p* = 0.020) (Table 1).

**Table 1:** Summary descriptive table of the patients included in the study by groups of sex.

Variables	All	Females	Males	*p*-value
	*N* = 374	*N* = 114	*N* = 260	
Age (years)	66.8 (9.65)	64.0 (10.2)	68.1 (9.16)	<0.001
BMI, kg/m^2^	26.1 (4.93)	25.1 (5.49)	26.5 (4.61)	0.018
HbA1c (%)	6.25 (0.90)	6.12 (0.80)	6.30 (0.94)	0.051
Total cholesterol (mg/dL)	180 (38.0)	196 (28.8)	173 (39.3)	<0.001
Urea (mg/dL)	41.6 (18.7)	37.3 (13.5)	43.5 (20.4)	0.001
Creatinine (mg/dL)	0.95 (0.77)	0.74 (0.32)	1.04 (0.88)	<0.001
Glomerular filtration (mL/min/1.73 m^2^)	81.9 (20.2)	85.2 (13.8)	80.4 (22.3)	0.012
Proteins (mg/dL)	6.97 (0.58)	6.96 (0.55)	6.98 (0.59)	0.716
Prealbumin (mg/dL)	23.3 (5.88)	22.7 (6.82)	23.6 (5.41)	0.209
Albumin (mg/dL)	3.96 (0.57)	3.90 (0.57)	3.99 (0.57)	0.132
C reactive protein (mg/dL)	35.2 (59.4)	30.0 (41.0)	37.5 (65.9)	0.187
TSH (μUI/mL)	1.83 (1.11)	2.02 (1.10)	1.75 (1.11)	0.032
ECOG:				0.650
0	153 (41.4%)	52 (45.6%)	101 (39.5%)	
1	153 (41.4%)	42 (36.8%)	111 (43.4%)	
2	53 (14.3%)	17 (14.9%)	36 (14.1%)	
3–4	11 (2.97%)	3 (2.63%)	8 (3.12%)	
Cancer type:				0.049
CPNCP	283 (76.1%)	84 (73.7%)	199 (77.1%)	
Others	13 (3.49%)	9 (7.89%)	4 (1.55%)	
CPCP	30 (8.06%)	7 (6.14%)	23 (8.91%)	
Mesothelioma	9 (2.42%)	2 (1.75%)	7 (2.71%)	
NP	37 (9.95%)	12 (10.5%)	25 (9.69%)	
TNM:				0.083
I	29 (11.9%)	6 (8.33%)	23 (13.4%)	
II	18 (7.38%)	6 (8.33%)	12 (6.98%)	
III	25 (10.2%)	4 (5.56%)	21 (12.2%)	
IVa	70 (28.7%)	17 (23.6%)	53 (30.8%)	
IVb	25 (10.2%)	7 (9.72%)	18 (10.5%)	
IVc	77 (31.6%)	32 (44.4%)	45 (26.2%)	
Chemotherapy				0.317
No	140 (40.1%)	46 (44.7%)	94 (38.2%)	
Yes	209 (59.9%)	57 (55.3%)	152 (61.8%)	
Radiotherapy				0.009
No	212 (60.4%)	73 (71.6%)	139 (55.8%)	
Yes	139 (39.6%)	29 (28.4%)	110 (44.2%)	
Immunotherapy				0.365
No	277 (80.5%)	77 (77.0%)	200 (82.0%)	
Yes	67 (19.5%)	23 (23.0%)	44 (18.0%)	
Complications				0.289
Absence	216 (57.8%)	71 (62.3%)	145 (55.8%)	
Presence	158 (42.2%)	43 (37.7%)	115 (44.2%)	
Exitus:				0.020
No	238 (63.6%)	83 (72.8%)	155 (59.6%)	
Yes	136 (36.4%)	31 (27.2%)	105 (40.4%)	

Data are represented as mean (SD) or n (%). Groups were divided by sex. A Shapiro–Wilks test was used to determine the normality of the variables. Asterisk indicates significant differences between groups according to the ANOVA or Kruskal test and further pairwise comparison of *p*-values, corrected for multiple comparisons test was used for variables expressed as percentage (^***^p < 0.001, ^**^p < 0.01, ^*^p < 0.05). BMI, body mass index; GLIM, The Global Leadership Initiative on Malnutrition; HbA1c, hemoglobin glycosylated; TSH, thyroid-stimulating hormone.

To assess the muscle quality and quantity of the patients included in this study, an ultrasound measurement of abdominal and leg muscle was conducted (Table 2). Significant sex-based differences were observed in nearly all nutritional ultrasound measurements, except for VAT (*p* = 0.956). Males had significantly greater rectus femoris muscle parameters, including RF-CSA, RF-CIRC, RF-*X*-axis, and RF-*Y*-axis, compared to females (*p* < 0.001 for all). RF-cont was also higher in males (*p* = 0.001). In contrast, females showed significantly higher levels of subcutaneous fat. Specifically, L-SAT, T-SAT, and S-SAT were all significantly greater in women than in men (*p* < 0.001 for all). GAT was markedly higher in females (*p* < 0.001 for both).

**Table 2:** Summary descriptive table of nutritional measurements of the patients included in the study by groups of sex.

Variables	All	Females	Males	p.overall
	*N* = 374	*N* = 114	*N* = 260	
RF-CSA, cm^2^	3.89 (1.36)	3.17 (1.19)	4.20 (1.31)	<0.001
RF-CIRC, cm	9.08 (1.27)	8.33 (1.22)	9.41 (1.15)	<0.001
RF-*X*-axis, cm	3.74 (0.82)	3.42 (0.63)	3.87 (0.86)	<0.001
RF-*Y*-axis, cm	1.15 (0.36)	1.02 (0.37)	1.20 (0.35)	<0.001
RF-cont, cm	1.71 (0.79)	1.49 (0.83)	1.81 (0.75)	0.001
L-SAT, cm	0.80 (0.45)	1.11 (0.46)	0.66 (0.37)	<0.001
T-SAT, cm	1.46 (0.66)	1.75 (0.75)	1.33 (0.58)	<0.001
S-SAT, cm	0.64 (0.36)	0.82 (0.42)	0.57 (0.29)	<0.001
VAT, cm	0.60 (0.36)	0.60 (0.34)	0.60 (0.37)	0.956
GAT, cm	2.85 (1.10)	3.46 (1.18)	2.59 (0.95)	<0.001

Data are represented as mean (SD) or *n* (%). Groups were divided by sex. A Shapiro–Wilks test was used to determine the normality of the variables. Asterisk indicates significant differences between groups according to the ANOVA or Kruskal test and further pairwise comparison of *p*-values, corrected for multiple comparisons test was used for variables expressed as percentage (^***^p < 0.001, ^**^p < 0.01, ^*^p < 0.05). Cont, Contraction; GAT, Global adipose tissue; GATi, Global adipose tissue index; RF-CIR, circumference of quadriceps rectus femoris; RF-CSA, rectus femoris cross-sectional area; SAT, subcutaneous adipose fat of leg (L), superficial (S) and total (T) abdominal.

### 3.2 Random forest to select the most important variables from the ultrasound measurement to predict malnutrition and muscle loss

Supplementary Table S1 presents the age- and BMI-adjusted ORs for associations between ultrasound-derived muscle and adipose tissue parameters and several nutritional and muscle-related conditions, including malnutrition (GLIM criteria), PG-SGA malnutrition, sarcopenia, dynapenia, and muscle mass status. The RF-CSA showed consistent and significant inverse associations with GLIM-defined malnutrition (OR = 0.63; 95% CI: 0.50–0.80), PG-SGA (OR = 0.69; 95% CI: 0.55–0.87), and most dynapenia-related variables. Similarly, RF-CIRC, RF-*Y*-axis, and RF-cont were inversely associated with GLIM, PG-SGA, and several dynapenia-related variables, all with statistically significant ORs below 1. Of note, the RF-*Y*-axis showed particularly strong inverse associations, especially for GLIM malnutrition and dynapenia-related variables, while RF-cont also showed significant associations with multiple outcomes. Regarding adiposity parameters, VAT was significantly associated with GLIM, although it’s wide confidence intervals in other conditions. The agreement analysis between GLIM and PG-SGA showed a moderate level of agreement (weighted *κ* = 0.51; 95% CI: 0.44–0.58; *n* = 374), while the unweighted kappa was 0.28 (95% CI: 0.22–0.35). These results suggest that both tools capture overlapping but not identical dimensions of malnutrition

To select the most important variable to assess both malnutrition and muscle quality and quantity, a Random Forest Analysis was conducted. Figure 1 displays the top three most important features identified by three separate random forest models predicting malnutrition, nutritional risk (PG-SGA), and dynapenia. Muscle ultrasound-derived variables consistently emerged among the most relevant predictors across all models. For malnutrition, the top predictors were RF-*Y*-axis, RF-CSA, VAT, RF-cont, and RF-CIR, followed by T-SAT, S-SAT, and RF-*X*-axis. This highlights the importance of both muscle thickness and central fat distribution in identifying malnourished patients (Figure 1A). In the PG-SGA model, the most influential variables were RF-Cont, RF-*Y*-axis, RF-CIR, RF-CSA, RF-*X*-axis, T-SAT, S-SAT, and VAT. These results suggest that both muscle dimension and fat reserves are key elements in determining nutritional risk as assessed by PG-SGA (Figure 1B). Finally, in the model predicting dynapenia, the leading variables were RF-CSA, RF-CIR, RF-*Y*-axis, and RF-Cont, followed by RF-*X*-axis, T-SAT, VAT, and S-SAT, reinforcing the role of muscle mass and size as central markers of reduced muscle strength (Figure 1C).

**Figure 1:**
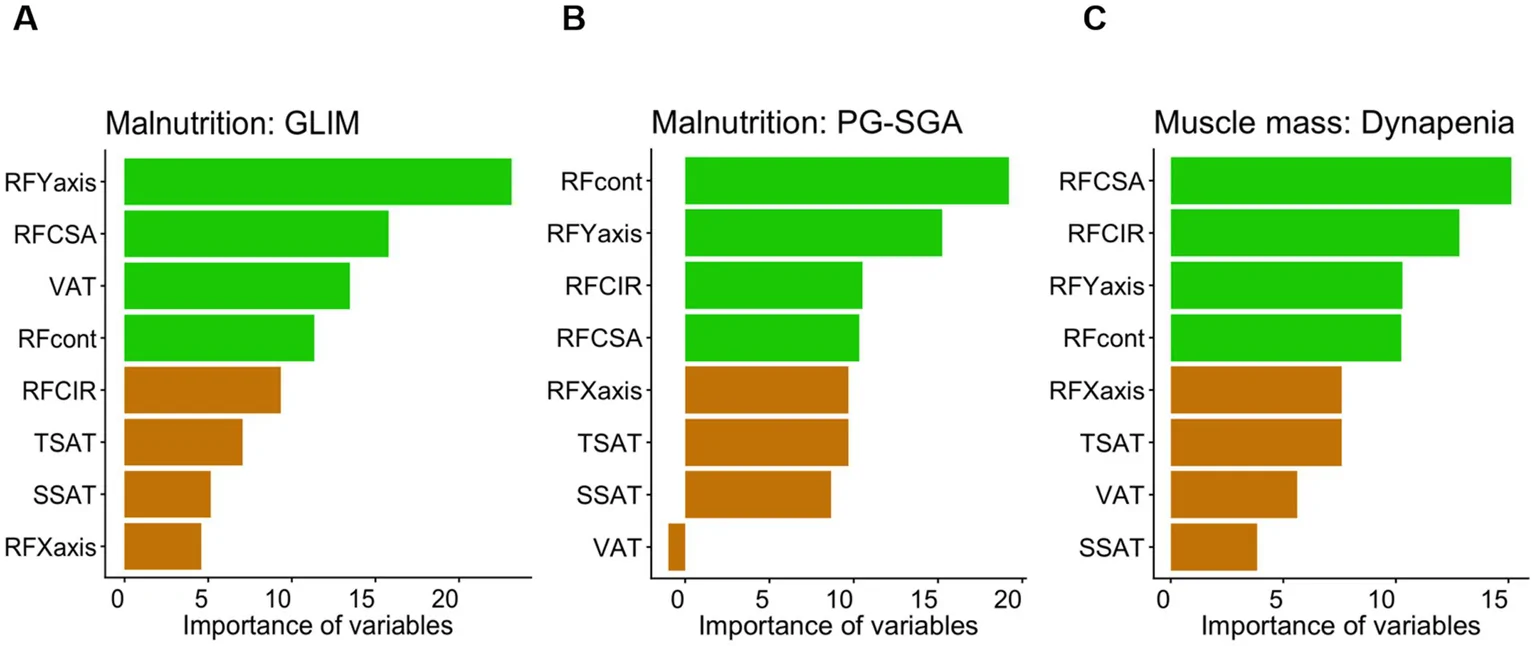
Variable importance plots from Random Forest models. **(A)** Variable importance for GLIM-defined malnutrition. **(B)** Variable importance for nutritional status assessed by PG-SGA. **(C)** Variable importance for dynapenia. Variable importance ranking was based on mean decrease in Gini index. The most relevant predictors include muscle ultrasound measurements. Higher values indicate greater importance in classification performance.

### 3.3 Discriminative capacity of muscle ultrasound variables for malnutrition from GLIM criteria

Table 3 summarizes the sex-stratified discriminative performance of muscle ultrasound measurements in detecting malnutrition according to GLIM criteria. Among the evaluated parameters, RF-CSA showed high discriminative ability in women (AUC = 0.814, sensitivity = 0.907, specificity = 0.650, *p* < 0.001) and moderate discriminative ability in men (AUC = 0.706, sensitivity = 0.729, specificity = 0.629, *p* < 0.001). RF-CIR also demonstrated statistically significant discriminatory performance in women (AUC = 0.738, sensitivity = 0.851, specificity = 0.666, *p* < 0.001) and men (AUC = 0.681, sensitivity = 0.597, specificity = 0.681, *p* < 0.001). RF-*Y*-axis showed AUCs of 0.804 in women and 0.713 in men, with sensitivities of 0.759 and 0.791 and specificities of 0.716 and 0.577, respectively (*p* < 0.001 for both). RF-*X*-axis showed lower but statistically significant discriminatory performance, with AUCs of 0.692 in women and 0.618 in men (*p* = 0.001 for both).

**Table 3:** Discriminative performance of muscle and body composition parameters for detecting malnutrition (GLIM).

Variables	AUC	Cut-off	Sensitivity	Specificity	*p*-value
RF-CSA					
Men	0.688	3.48	0.574	0.771	<0.001^***^
Women	0.787	2.97	0.636	0.846	<0.001^***^
RF-CIR					
Men	0.653	9.42	0.598	0.688	<0.001^***^
Women	0.750	8.00	0.769	0.772	<0.001^***^
RF-*X*-axis					
Men	0.592	4.16	0.289	0.901	<0.001^***^
Women	0.722	3.46	0.613	0.808	0.001^**^
RF-*Y*-axis					
Men	0.705	0.91	0.893	0.491	<0.001^***^
Women	0.807	0.78	0.875	0.576	<0.001^***^

Area under the curve (AUC), optimal cut-off values, sensitivity, specificity, and *p*-values are shown for each variable, stratified by sex, in distinguishing between well-nourished vs. malnourished individuals. Significant variables (*p* < 0.05) indicate good discriminatory capacity.

### 3.4 Association between muscle ultrasound parameters and adverse outcomes

Table 4 presents the associations between muscle ultrasound measurements and the risk of mortality and complications, stratified by sex and adjusted for age, BMI, ECOG performance status, cancer type, and cancer stage. In men, RF-CSA was not significantly associated with mortality (HR = 0.84, 95% CI: 0.69–1.02), but it was associated with lower odds of complications (OR = 0.79, 95% CI: 0.60–1.00); no significant associations were observed in women. Similarly, RF-CIR was not significantly associated with mortality in men (HR = 0.86, 95% CI: 0.71–1.06), although it was associated with lower odds of complications (OR = 0.73, 95% CI: 0.56–0.94), whereas no significant associations were found in women. RF-*Y*-axis was significantly inversely associated with mortality in men (HR = 0.32, 95% CI: 0.15–0.71), but not with complications (OR = 0.45, 95% CI: 0.16–1.21), and no significant associations were observed in women. The RF-*X*-axis was also negatively associated with complications in men (OR = 0.61, 95% CI: 0.37–0.94), although it was not associated with mortality.

**Table 4:** Association between muscle composition parameters and mortality risk, stratified by sex.

Variables	Mortality	Complications
	HR (CI 95%)	OR (CI 95%)
RF-CSA		
Men	0.84 (0.69–1.02)	0.79 (0.60–1.00)^*^
Women	1.06 (0.72–1.56)	0.74 (0.43–1.34)
RF-CIR		
Men	0.86 (0.71–1.06)	0.73 (0.56–0.94)^*^
Women	0.92 (0.64–1.32)	0.90 (0.60–1.34)
RF-*X*-axis		
Men	1.07 (0.78–1.45)	0.61 (0.37–0.94)^*^
Women	0.66 (0.34–1.30)	1.18 (0.57–2.52)
RF-*Y*-axis		
Men	0.32 (0.15–0.71)^**^	0.45 (0.16–1.21)
Women	1.28 (0.41–3.94)	0.53 (0.13–2.00)

Hazard ratios (HR) and 95% confidence intervals (CI) for all-cause mortality are shown for each variable, based on sex-specific cut-off values. Models were adjusted for age, BMI, ECOG, caner type and cancer stage. ECOG performance status was categorized as 0–1 versus ≥2, tumor stage as I–II versus III–IV, and tumor type as NSCLC, SCLC, and Other thoracic malignancies. Significant associations (*p* < 0.05) are indicated with an asterisk (^*^).

To evaluate the understanding of these results, a decision tree and a Kaplan–Meier analysis were conducted. In Figure 2, decision tree analysis was conducted to identify muscle ultrasound cut-off points that could effectively discriminate survival outcomes by sex. In men, the algorithm selected a RF-*Y*-axis threshold of 0.77 cm, which was able to separate patients into high and low mortality risk groups. This cut-off significantly discriminated survival in the Kaplan–Meier analysis, with patients below the threshold showing significantly higher mortality (*p* < 0.001). In women, the model also identified a RF-*Y*-axis threshold of 0.6 cm as the optimal discriminator. Similarly, women with RF-*Y*-axis measurements below this cut-off had significantly worse survival outcomes compared to those above the threshold, as confirmed by the Kaplan–Meier curves (*p* = 0.0013).

**Figure 2:**
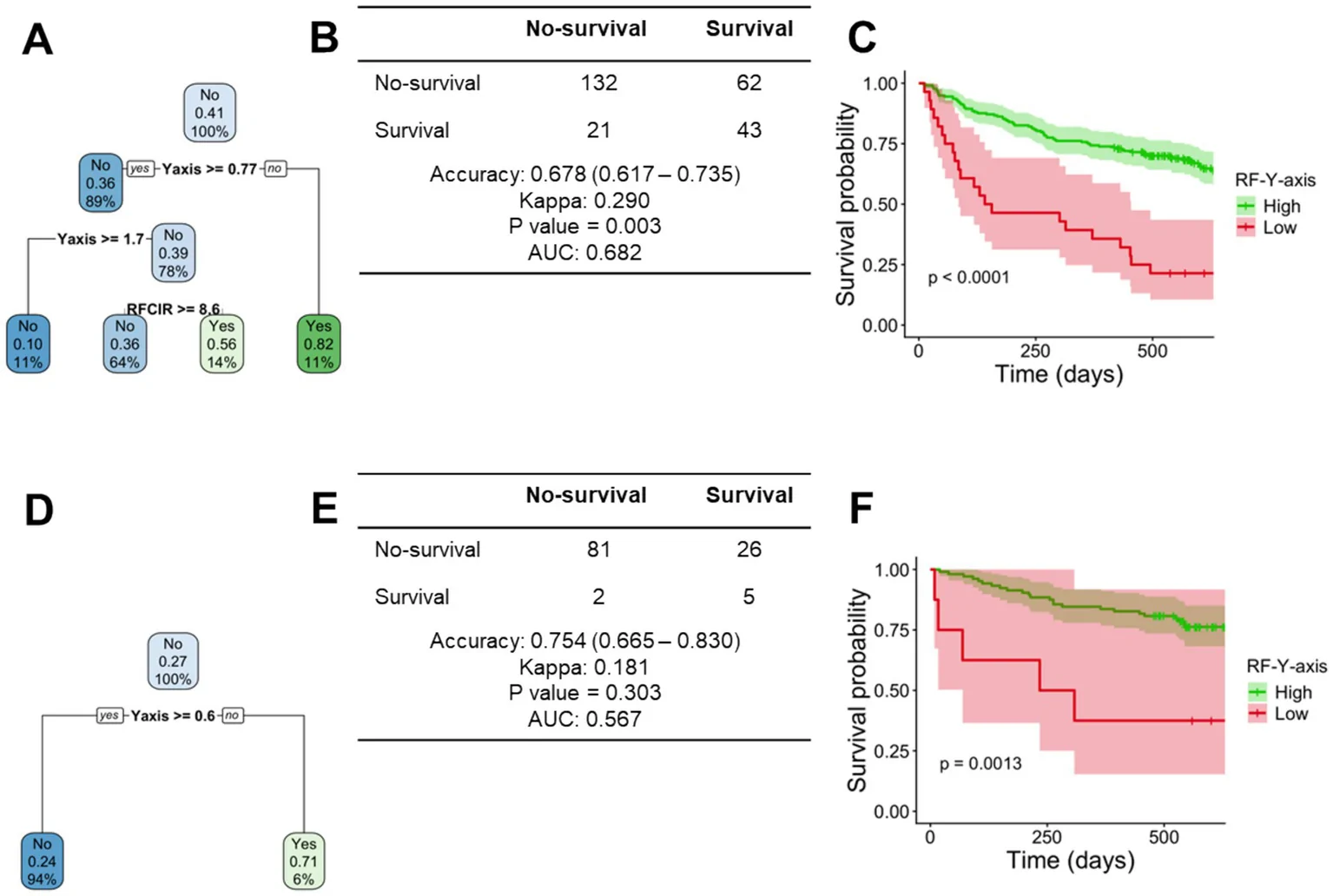
Decision tree-based risk stratification using ultrasound parameters in men and women and Kaplan–Meier survival curves according to baseline nutritional ultrasound values (low vs. high). **(A)** Shows the decision tree for male patients, identifying rectus femoris thickness along the *Y*-axis as the main predictor of mortality, with a cut-off of 0.77 cm. **(B)** Presents the confusion matrix for the male model, supporting its classification performance. **(C)** Displays the Kaplan–Meier survival analysis in men stratified by the 0.77 cm cut-off, with significantly poorer survival below this threshold (log-rank *p* < 0.001). **(D)** Illustrates the decision tree for female patients, where rectus femoris thickness along the *Y*-axis was the principal predictor, with a cut-off of 0.6 cm. **(E)** Reports the confusion matrix for the female model, summarizing its classification accuracy. **(F)** Shows the Kaplan–Meier survival curves in women based on the cut-off, revealing significantly different survival outcomes between groups (*p* = 0.0013).

## 4 Discussion

The present multicenter study suggests for the first time in a large lung cancer cohort that selected rectus femoris ultrasound-derived parameters are associated with mortality and clinical complications. Beyond their association with malnutrition and dynapenia, muscle ultrasound measurements showed sex-specific associations with clinical outcomes, particularly in men, supporting the clinical relevance of morphofunctional assessment in this population. Notably, 60 to 80% of patients with advanced cancer suffer from disease-related malnutrition (22), and sarcopenia in lung cancer is associated with an approximately threefold higher mortality risk, regardless of disease stage (13, 23). To address this critical issue, a large cohort of 374 patients with baseline characteristic comparable to smaller previous studies was analyzed (11, 13, 24–26). It should be noted that even the combined sample sizes of the previously reported small cohorts that applied morphofunctional nutritional assessment, including nutritional ultrasound, did not reach that of the present study, which therefore provides greater statistical power and enhances the external validity of the clinical findings.

Cancer cachexia and sarcopenia are frequently observed in individuals with lung cancer and have been consistently associated with an increased incidence of postoperative complications, reduced tolerance to antineoplastic therapies, and poorer overall survival (20, 27–29). From a clinical and nutritional perspective, the early recognition of these conditions may be critical for optimizing patient management, enabling timely nutritional interventions and the implementation of more individualized therapeutic approaches.

Consistent with most previous reports, the present cohort showed a higher prevalence of lung cancer among men. However, no significant sex-related differences were observed in ECOG performance status or disease stage, whereas cancer type differed significantly between sexes as previously reported (11, 13, 26, 30).

Significant sexual dimorphism was observed in muscle and adipose compartments by nutritional ultrasonography. Men exhibited larger rectus femoris dimensions (CSA, circumference, transverse and anteroposterior diameters), whereas women showed higher subcutaneous adipose thickness despite similar visceral fat values. These findings align with the multicenter DRECO study (31), which established sex-specific ultrasound cut-offs for rectus femoris CSA and thickness in nearly 1,000 hospitalized patients at nutritional risk, reporting CSA thresholds between 2.4 and 3.66 cm^2^ and *Y*-axis values between 0.785 and 1.04 cm, consistently higher in men. Prior oncological evidence by López-Gómez et al. (23). further demonstrated significant correlations between CSA and anthropometry, phase angle, and handgrip strength, supporting the construct validity of muscle ultrasound in cancer populations. The present findings are consistent with previous morphofunctional ultrasound studies. Importantly, the clinical applicability of these measurements is further extended to lung cancer, where sex-specific muscle thresholds demonstrated both diagnostic relevance and sex-specific associations with mortality and clinical complications, mainly in men. These findings reinforce the need for sex-adjusted reference standards, as non-specific cut-offs may reduce sensitivity for detecting clinically meaningful muscle impairment, particularly in women with lower absolute muscle mass and higher adiposity. Collectively, this evidence supports interpreting muscle measurements within sex-normative frameworks (16, 32) and highlights the potential clinical relevance of bedside ultrasound for integrated muscle–adipose assessment. Although CT remains the gold standard, strong agreement between ultrasound-derived adipose measures and CT-based indices (11), supports ultrasound as a valid and feasible body composition tool.

In the same way, logistic regression showed that ultrasound-derived muscle parameters were significantly associated with malnutrition and dynapenia in lung cancer. Reduced rectus femoris CSA and circumference were significantly associated with higher odds of malnutrition according to both GLIM and PG-SGA criteria, as well as with dynapenia, after adjustment for age and BMI. Reduced rectus femoris thickness along the *Y*-axis emerged as a particularly relevant marker, with lower values associated with higher odds of GLIM-defined malnutrition and dynapenia. These relationships suggest that losses in muscle mass and thickness are tightly associated with worsening nutritional status and functional decline, as expected within this clinical setting (11, 29). Rectus femoris thickness and cross-sectional area were consistently found to be important predictors of both malnutrition and impaired muscle function by the Random Forest analysis, supporting the central role of muscle wasting in these conditions (33, 34). This pattern makes biological sense because cachexia, a condition in which systemic inflammation speeds up muscle loss and functional decline, often coexists with malnutrition in lung cancer (30). The present results are in agreement with prior reports showing that patients fulfilling malnutrition criteria often present with concurrent sarcopenia and frailty (23, 24, 26). In this regard, Wang et al. demonstrated in hospitalized patients with lung cancer that GLIM-defined malnutrition was strongly associated with both sarcopenia and frailty, indicating that insufficient intake and inflammatory burden manifest clinically through combined weight loss and muscle dysfunction (26). Similarly, Lopez-Gómez et al. found that ultrasound-measured rectus femoris area was significantly lower in cancer patients with sarcopenia, mirroring our observation that ultrasound metrics distinguish those with muscle depletion (23). The link between dynapenia and low ultrasound muscle measures is also expected, since reduced muscle thickness/quality directly translates to reduced strength. Cancer cachexia often involves not just quantitative muscle loss but qualitative changes like fatty infiltration and fibrosis, which impair muscle function (34, 35).

Similarly, the incorporation of muscle ultrasound may strengthen the discriminative capacity of the GLIM framework. The GLIM criteria rely in part on subjective clinical evaluation of muscle loss, a method that often lacks precision and may lead to under-diagnosis of malnutrition (26). In contrast, ultrasound provides an objective and quantitative assessment of muscle deficits, thereby supporting a more standardized evaluation of muscle loss. Among the parameters evaluated, rectus femoris CSA and *Y*-axis thickness emerged as the most discriminative markers, displaying the highest AUCs in both sexes. In head and neck cancer, a recent multicenter study identified an RF-CSA cut-off of 2.7 cm^2^ with an AUC of 0.653 for mortality prediction and demonstrated independent associations with GLIM-defined malnutrition and sarcopenia. In that cohort, higher RF-CSA and *Y*-axis values were significantly associated with lower odds of malnutrition (OR 0.81 and 0.31, respectively) and sarcopenia (OR 0.64 and 0.27), reinforcing their diagnostic robustness (15). Other measures, such as muscle circumference and *X*-axis width, demonstrated lower discriminatory performance, reinforcing that CSA and *Y*-axis appeared to be the most informative ultrasound indicators in this analysis. In the present lung cancer cohort, comparable discriminatory patterns were observed; however, beyond diagnostic performance, selected ultrasound parameters showed sex-specific adjusted associations with mortality and clinical complications, particularly in men, with RF-*Y*-axis associated with mortality and RF-CSA, RF-CIR, and RF-*X*-axis associated with complications. These results have several clinically relevant implications. First, the analysis confirms the necessity of sex-specific diagnostic thresholds. Optimal cut-offs differed between men and women, reflecting inherent differences in baseline muscle mass. This observation is consistent with previous recommendations highlighting the need for sex- and population-specific criteria (16, 32). Importantly, applying sex-adjusted cut-offs may improve sensitivity in women, who are at risk of being under-diagnosed when male-oriented thresholds are used.

Second, ultrasound may meaningfully enhance the practical implementation of the GLIM muscle criterion. Although CT and BIA have traditionally been used to assess muscle mass, their availability, cost, and technical limitations restrict routine application in oncology settings (26, 30). Recent work combining CT and functional tests has shown improved sarcopenia detection (11), and the present findings indicate that ultrasound can a feasible complementary assessment without the need for CT imaging.

Third, these data support the potential integration of ultrasound into standard nutritional screening in oncology. Conventional tools such as NRS-2002 or MST focus on weight loss and intake but do not directly assess body composition, which may delay recognition of malnutrition (32). Ultrasound-derived muscle metrics, particularly CSA and *Y*-axis, showed measurable discriminatory ability for detecting GLIM-defined malnutrition, with higher AUCs than the other rectus femoris parameters evaluated. This aligns with growing evidence that bedside ultrasound (POCUS: Point Of Care Ultrasound) offers a practical, objective complement to traditional screening (16).

The results indicate that selected ultrasound-derived muscle measurements are significant markers associated with clinical outcomes, including mortality and clinical complications, with clear sex-specific differences (Table 4). In male patients, reduced rectus femoris *Y*-axis thickness was associated with higher mortality risk, whereas RF-CSA, RF-CIR, and RF-*X*-axis were associated with clinical complications; these associations were not significant in women. This finding suggests that insufficient muscle depth, reflecting severe sarcopenia, represents a critical prognostic marker particularly in men, although the relevant risk thresholds appear to differ between men and women. This sex-specific pattern is likely related to underlying anatomical and compositional differences in muscle distribution between sexes. This interpretation is supported by previous evidence showing that sex-specific cut-offs improve risk stratification. To mention, García-García et al. (32) found that a rectus femoris thickness below 1.0 cm in women and 1.06 cm in men was predictive of poorer survival, and low muscle CSA cut-offs also stratified risk by sex. The present data are consistent with previous evidence and further extend it by suggesting that selected muscle ultrasound parameters may be prognostically relevant for mortality and complications in lung cancer. From a clinical perspective, these findings have important implications for prognostic stratification and personalized care. Specifically, a simple ultrasound assessment of the rectus femoris may help identify patients at high risk of adverse outcomes, even when conventional indicators such as weight or BMI appear unremarkable. Thus, patients with markedly reduced rectus femoris dimensions at diagnosis can be flagged as vulnerable, prompting earlier nutritional support, physical rehabilitation, or closer monitoring during oncologic treatment (36). Previous studies have already shown that altered nutritional status is associated with poorer treatment tolerance in lung cancer (25, 26). In this context, muscle ultrasound markers may offer complementary and clinically meaningful prognostic information, with the advantage of directly reflecting skeletal muscle mass and functional reserve, which were significantly associated with functional decline, prolonged hospitalization, or reduced survival (16). The current data add specific evidence in lung cancer, suggesting that selected ultrasound-derived muscle parameters are associated with increased mortality and morbidity, mainly in men, thereby reinforcing the rationale for integrating muscle ultrasound into routine oncologic assessment.

Moreover, the multicenter nature of our study, involving a total of 13 tertiary hospitals and encompassing 374 patients, reinforces the generalizability of our findings. Nevertheless, several limitations must be acknowledged. The observational design precludes causal inference, and ultrasound is an operator-dependent technique; although measurements were performed by trained personnel, the absence of reported intra- and interobserver reproducibility indicators may limit generalizability without adequate staff training and standardized acquisition protocols. Additionally, the assessment focused on muscle quantity at a single anatomical site, which, although validated, may not fully capture interindividual variability in muscle loss across different muscle groups.

## 5 Conclusion

The findings of this multicenter observational study show that ultrasound-based assessment of the quadriceps, with a focus on the rectus femoris, provides clinically relevant information in lung cancer patients. Rectus femoris dimensions and thickness parameters revealed clear sex-specific patterns and were associated with nutritional status, muscle performance, and unfavorable clinical outcomes, such as mortality and clinical complications, particularly in men. From a clinical and nutritional perspective, these results imply that rectus femoris ultrasound could be an objective, bedside method that supports earlier and more precise risk stratification beyond traditional anthropometric measurements and supplements well-established frameworks like GLIM. Future prognostic models in lung cancer may benefit from integrating muscle ultrasound with functional evaluations and complementary body composition techniques.

There are some limitations that should be considered. Causal relationships cannot be established because the study is observational in nature. Because ultrasound evaluations were carried out by skilled operators, their applicability in situations without standardized training procedures may be limited. Moreover, ultrasound is an operator-dependent technique, and the absence of reported intra- and interobserver reproducibility indicators should be considered when interpreting the multicenter applicability of these measurements. In addition, although the study was approved by the local ethics committee before initiation, it was not prospectively registered in a public clinical or observational study registry, which should be considered when interpreting the reporting framework of the study. Furthermore, the assessment was limited to a single muscle site and focused mainly on muscle quantity without considering ultrasound markers of muscle quality. The longitudinal trajectories of muscle ultrasound parameters during oncological treatment, the confirmation of these findings in separate cohorts, and the determination of whether interventions based on ultrasound findings result in meaningful improvements in clinical outcomes all require additional research.

## Data Availability

The datasets presented in this study can be found in online repositories. The names of the repository/repositories and accession number(s) can be found in the article.
