# Supplementary material for: Nutritional ultrasound cut off for predicting complications and mortality in lung cancer patients: a multicenter longitudinal study from the VALONC project
Source: Front Nutr. 2026 Sep 17;13:1876352. doi: 10.3389/fnut.2026.1876352 (PMC13629502; doi:10.3389/fnut.2026.1876352)
Supplement: Supplementary file 1 [file Supplementary_file_1.docx]

|  | **GLIM** | **PG-SGA** | **Sarcopenia** | **Dynapenia** | **Dynapenia “Pizarra”** | **Low muscle mass** | **Dynapenia without muscle loss** |
| --- | --- | --- | --- | --- | --- | --- | --- |
| **Variables** | OR (CI 95%) | OR (CI 95%) | OR (CI 95%) | OR (CI 95%) | OR (CI 95%) | OR (CI 95%) | OR (CI 95%) |
| RF-CSA, cm^2^ | 0.69 (0.51 - 0.90)** | 0.69 (0.55 - 0.87)** | 0.31 (0.13 - 0.64)** | 0.55 (0.41 - 0.73)*** | 0.7 (0.54 - 0.89)** | 0.72 (0.53 - 0.96)* | 0.85 (0.46 - 1.55) |
| RF-CIRC, cm | 0.72 (0.56 - 0.92)* | 0.7 (0.53 - 0.91)** | 0.51 (0.3 - 0.85)* | 0.66 (0.51 - 0.85)** | 0.75 (0.59 - 0.95)* | 0.71 (0.53 - 0.93)* | 0.71 (0.38 - 1.24) |
| RF-X-axis, cm | 0.71 (0.45 - 1.05) | 0.91 (0.66 - 1.31) | 0.99 (0.54 - 1.57) | 0.77 (0.49 - 1.12) | 0.84 (0.56 - 1.16) | 0.72 (0.43 - 1.1) | 0.62 (0.28 - 1.71) |
| RF-Y-axis, cm | 0.34 (0.11 - 0.96)* | 0.28 (0.11 - 0.67)** | 0.03 (0 - 0.37)** | 0.2 (0.07 - 0.56)** | 0.34 (0.12 - 0.87)* | 0.27 (0.08 - 0.85)* | 1.17 (0.13 - 11.13) |
| RF-cont, cm | 0.63 (0.35 - 1.01) | 0.48 (0.32 - 0.69)*** | 0.11 (0.01 - 0.56)* | 0.41 (0.19 - 0.77)* | 0.97 (0.65 - 1.34) | 0.61 (0.34 - 1.02) | 1.48 (0.47 - 5.34) |
| L-SAT, cm | 1.01 (0.4 - 2.51) | 0.82 (0.38 - 1.83) | 3.05 (0.44 - 22.3) | 2.58 (1.12 - 5.97)* | 1.58 (0.69 - 3.57) | 0.91 (0.31 - 2.61) | 0.8 (0.07 - 9.49) |
| T-SAT, cm | 0.5 (0.27 - 0.88)* | 1.06 (0.65 - 1.75) | 2.57 (0.78 - 8.61) | 0.82 (0.48 - 1.38) | 0.76 (0.45 - 1.25) | 1.34 (0.74 - 2.44) | 2.44 (0.7 - 9.89) |
| S-SAT, cm | 0.54 (0.18 - 1.50) | 2.49 (1 - 6.91) | 3.07 (0.41 - 21.45) | 1.68 (0.67 - 3.99) | 1.26 (0.52 - 2.93) | 1.44 (0.47 - 4.17) | 7.97 (0.63 - 145.08) |
| VAT, cm | 0.67 (0.26 - 1.58) | 0.56 (0.28 - 1.14) | 2.26 (0.34 - 10.18) | 0.95 (0.38 - 2.12) | 1.54 (0.74 - 3.1) | 0.7 (0.25 - 1.73) | 6.85 (0.42 - 172) |
| GAT, cm | 0.7 (0.48 - 1.01) | 0.88 (0.63 - 1.22) | 1.97 (0.93 - 4.32) | 1.06 (0.75 - 1.49) | 1.04 (0.75 - 1.43) | 1.05 (0.71 - 1.54) | 2.19 (0.8 - 7.13) |
|  |  |  |  |  |  |  |  |

**Table S1**. Odds Ratios for malnutrition and muscle loss According to Ultrasound measurement

This table shows the results of logistic regression analysis assessing the association between nutritional status and various functional variables using ultrasound method. For each variable, odds ratios (OR) with 95% confidence intervals (CI) and p-values are reported. All analyses were adjusted by age and BMI. An OR > 1 indicates higher odds of malnutrition and muscle loss with increasing values, whereas an OR < 1 indicates lower odds. **p < 0.05, ** p < 0.01 and *** p < 0.001 considered statistically significant and is indicated by an asterisk (*) in the table **Abbreviations**. Cont: Contraction; GAT: Global adipose tissue, GATi: Global adipose tissue index; RF-CIR: circumference of quadriceps rectus femoris; RF-CSA: rectus femoris cross-sectional area; SAT: subcutaneous adipose fat of leg (L), superficial (S) and total (T) abdominal.
