# Supplementary material for: Nutritional ultrasound cut off for predicting complications and mortality in lung cancer patients: a multicenter longitudinal study from the VALONC project
Source: Front Nutr. 2026 Sep 17;13:1876352. doi: 10.3389/fnut.2026.1876352 (PMC13629502; doi:10.3389/fnut.2026.1876352)
Supplement: Supplementary file 2 [file Supplementary_file_2.docx]

"Create a high-resolution scientific graphical abstract for a peer-reviewed medical/nutrition journal, in a clean academic infographic style, with a pure white background, subtle pale-green panels, dark teal headings, thin divider lines, and realistic but simplified medical illustrations. Use a wide horizontal 16:9 layout divided into three equal vertical columns. The overall appearance should be professional, clinical, elegant, highly legible, and suitable for Frontiers in Nutrition. Avoid decorative elements, gradients, shadows, 3D effects, or clutter.

COLUMN 1 — “1. Clinical problem and study aim”

Place a simplified anatomical illustration of a patient with the lungs visible, showing a lung tumor and the quadriceps/rectus femoris highlighted.

Add the following text in clearly separated blocks:

Malnutrition and muscle depletion are common in lung cancer and may be missed by BMI or weight alone.

Muscle wasting is linked to treatment toxicity, complications and poorer survival.

At the bottom, create a pale-green rounded box with a target icon and the heading:

Aim

To evaluate whether bedside rectus femoris ultrasound can identify high-risk lung cancer patients and predict malnutrition, complications and 1-year mortality, and to determine sex-specific cut-offs.

COLUMN 2 — “2. Study methodology”

At the top, show a hospital icon and a group of male and female patient silhouettes.

Display prominently:

10 Spanish hospitals

374 lung cancer patients

Multicenter, longitudinal observational study

In the center, show a detailed medical illustration of the thigh with the rectus femoris (quadriceps) highlighted, an ultrasound probe positioned over the muscle, and an ultrasound monitor showing a transverse rectus femoris image.

Label the ultrasound image with arrows for:

X-axis

Y-axis

Add:

Rectus femoris (quadriceps) assessed by bedside ultrasound.

Beside it, create a small pale-green box:

Key ultrasound variables:

• RF-CSA (cross-sectional area)
• RF-CIR (circumference)
• RF-X-axis (width)
• RF-Y-axis (thickness)

At the bottom, write:

Ultrasound data were related to GLIM malnutrition, PG-SGA, dynapenia, clinical complications and 1-year mortality.

Below this sentence, place four simple clinical icons with labels:

Malnutrition (GLIM, PG-SGA)
Dynapenia (low strength)
Clinical complications
1-year mortality

COLUMN 3 — “3. Key findings and clinical impact”

Divide this section into four clearly separated subsections labeled A, B, C and D using dark-green circular markers.

A. Sex differences

Use a blue male icon and a pink female icon.

Text:

Men had larger rectus femoris muscle dimensions.

Women had higher subcutaneous fat.

B. Best markers of malnutrition

Text:

RF-CSA and RF-Y-axis were the best discriminative ultrasound markers for GLIM-defined malnutrition.

Add a clean scientific table:

| Sex | RF-CSA (cm²) | RF-Y-axis (cm) |
| Men | 3.48 | 0.91 |
| Women | 2.97 | 0.78 |

Use a blue male symbol and pink female symbol in the first column.

C. Prognostic associations

Use small dark-green bullet points:

• In men, lower RF-Y-axis was associated with higher all-cause mortality (HR 0.32; 95% CI 0.15–0.71).
• In men, lower RF-CSA, RF-CIR and RF-X-axis were associated with more complications.
• No significant adjusted Cox associations were observed in women.

D. Decision-tree survival thresholds

Add a compact two-row table:

| Sex | RF-Y-axis (cm) |
| Men | < 0.77 cm → poorer survival |
| Women | < 0.60 cm → poorer survival |

Use the same blue male and pink female symbols.

Across the full bottom of the infographic, add a dark teal-green horizontal banner with a small clinical/lightbulb icon and the concluding sentence:

“Bedside rectus femoris ultrasound is a simple, objective, non-invasive tool that complements GLIM and supports early nutritional and prognostic risk stratification in lung cancer.”

Visual style requirements: white background; dark teal-green section headers; pale mint-green content boxes; blue for male icons; muted pink/magenta for female icons; black or very dark navy text; sans-serif typography similar to Arial, Helvetica, or Source Sans; realistic anatomical illustrations combined with clean vector medical icons; generous white space; precise alignment; consistent margins; no unnecessary decoration.

Critical requirement: reproduce all numerical values, labels, abbreviations and scientific terminology exactly as written above. Do not invent, alter or omit any data. Ensure every word is correctly spelled and fully readable. The final result must look like a professionally designed journal graphical abstract, not a presentation slide or commercial infographic."
